# Beyond Total PSA: Clinical Significance of S2,3PSA% in Reducing Unnecessary Prostate Biopsies

**DOI:** 10.1111/iju.70371

**Published:** 2026-02-03

**Authors:** Shingo Hatakeyama, Tohru Yoneyama, Chikara Ohyama

**Affiliations:** ^1^ Department of Urology Hirosaki University Graduate School of Medicine Hirosaki Japan; ^2^ Department of Glycotechnology Hirosaki University Graduate School of Medicine Hirosaki Japan; ^3^ Department of Advanced Transplant and Regenerative Medicine Hirosaki University Graduate School of Medicine Hirosaki Japan

**Keywords:** biomarker, biopsy, prostate cancer, prostate‐specific antigen, S2,3PSA%

## Abstract

Prostate‐specific antigen (PSA) testing has long been central to prostate cancer detection but is limited by poor specificity, resulting in overdiagnosis and unnecessary prostate biopsies. Although PSA derivatives such as percentage free PSA and the Prostate Health Index (PHI) have improved diagnostic performance, substantial uncertainty persists, particularly in patients with equivocal magnetic resonance imaging (MRI) findings. Cancer‐associated alterations in PSA glycosylation have emerged as a promising strategy to address these limitations. Percentage α2,3‐linked sialylated PSA (S2,3PSA%) reflects tumor‐associated glycoform changes that differ from those observed in benign prostatic conditions. This review summarizes the biological basis, analytical development, and clinical evidence supporting S2,3PSA% as a novel biomarker for prostate cancer diagnosis. We highlight key studies demonstrating that S2,3PSA% improves discrimination of clinically significant prostate cancer and provides complementary information when combined with PHI and MRI. Recent data further indicate that integrated diagnostic approaches incorporating S2,3PSA%, PHI, and PI‐RADS scoring can meaningfully reduce unnecessary prostate biopsies without compromising detection of clinically significant disease. Beyond diagnostic accuracy, emerging evidence suggests that S2,3PSA%–guided risk stratification in screening settings may also reduce the number of unnecessary MRI examinations and biopsies, thereby contributing to more efficient use of healthcare resources and potential cost savings. We also discuss the potential role of S2,3PSA% in prostate cancer screening and active surveillance. Collectively, current evidence supports S2,3PSA% as a biologically informed biomarker that helps reduce diagnostic uncertainty inherent to PSA‐based decision‐making and facilitates more individualized and resource‐conscious prostate cancer care.

## 1 Introduction

Prostate‐specific antigen (PSA) has been the cornerstone of prostate cancer detection for more than three decades [1, 2, 3, 4, 5]. While PSA‐based screening has contributed to earlier diagnosis and reduced prostate cancer–specific mortality, its limited specificity has resulted in substantial overdiagnosis and unnecessary prostate biopsies [6, 7, 8, 9, 10, 11, 12, 13, 14, 15]. Patients with moderately elevated PSA levels frequently undergo invasive biopsy procedures despite harboring no clinically significant prostate cancer (csPC). This diagnostic dilemma has persisted even with the introduction of PSA derivatives such as percentage free PSA (%fPSA), [−2]proPSA, and the Prostate Health Index (PHI) [16, 17, 18, 19, 20, 21, 22, 23, 24].

The advent of multiparametric magnetic resonance imaging (mpMRI) and MRI‐targeted biopsy has represented a paradigm shift in prostate cancer diagnostics. MRI‐based risk stratification using Prostate Imaging Reporting and Data System (PI‐RADS) has improved the detection of csPC and reduced random biopsies. However, MRI alone does not fully resolve diagnostic uncertainty. Equivocal lesions (PI‐RADS 3), false‐positive findings, and MRI‐negative yet clinically significant tumors remain challenging scenarios in daily practice [25, 26, 27, 28, 29, 30, 31, 32, 33, 34, 35, 36, 37, 38]. Consequently, the need for biologically complementary biomarkers that refine biopsy decision‐making in the MRI era remains unmet.

Recent advances in cancer glycobiology have revealed that alterations in glycosylation patterns are a hallmark of malignant transformation. PSA, a glycoprotein secreted by prostate epithelial cells, undergoes cancer‐associated changes in its glycan structures [20, 39, 40, 41, 42, 43, 44, 45]. Percentage α2,3‐linked sialylated PSA (S2,3PSA%) has emerged as a novel biomarker that captures these qualitative differences in PSA glycosylation rather than PSA concentration alone [43, 44, 45]. Accumulating evidence suggests that S2,3PSA% provides clinically relevant information that complements both conventional PSA derivatives and MRI findings [43, 45, 46].

This review comprehensively summarizes the biological rationale, analytical development, and clinical evidence supporting S2,3PSA% as a biomarker for prostate cancer diagnosis. Particular emphasis is placed on its role in reducing unnecessary prostate biopsies through integration with PHI and MRI‐based risk stratification.

## 2 Biological Basis of Cancer‐Associated PSA Glycosylation

### 2.1 PSA Glycoforms in Benign and Malignant Prostate Tissue

PSA is a kallikrein‐related serine protease that is extensively glycosylated. Glycosylation is not a random process; rather, it is tightly regulated by cellular enzymatic machinery and reflects the biological state of the producing cells. Early glycobiological studies demonstrated that PSA secreted from benign prostate tissue predominantly exhibits α2,6‐linked sialylation (S2,6PSA), whereas PSA derived from prostate cancer tissue is enriched with α2,3‐linked sialylation (S2,3PSA) [47, 48]. These cancer‐associated glycosylation changes are consistent with broader oncological observations in which α2,3‐sialylation (and the expression of α2,3‐sialylated glycans) is frequently increased during malignant transformation. Accumulating evidence indicates that increased sialylation can promote tumor invasion and metastatic behavior and may facilitate immune evasion through inhibitory Siglec–sialic acid interactions [49, 50]. Importantly, such glycan alterations occur independently of PSA concentration. This suggests that PSA glycoforms encode information that is distinct from PSA quantity.

**FIGURE 1:**
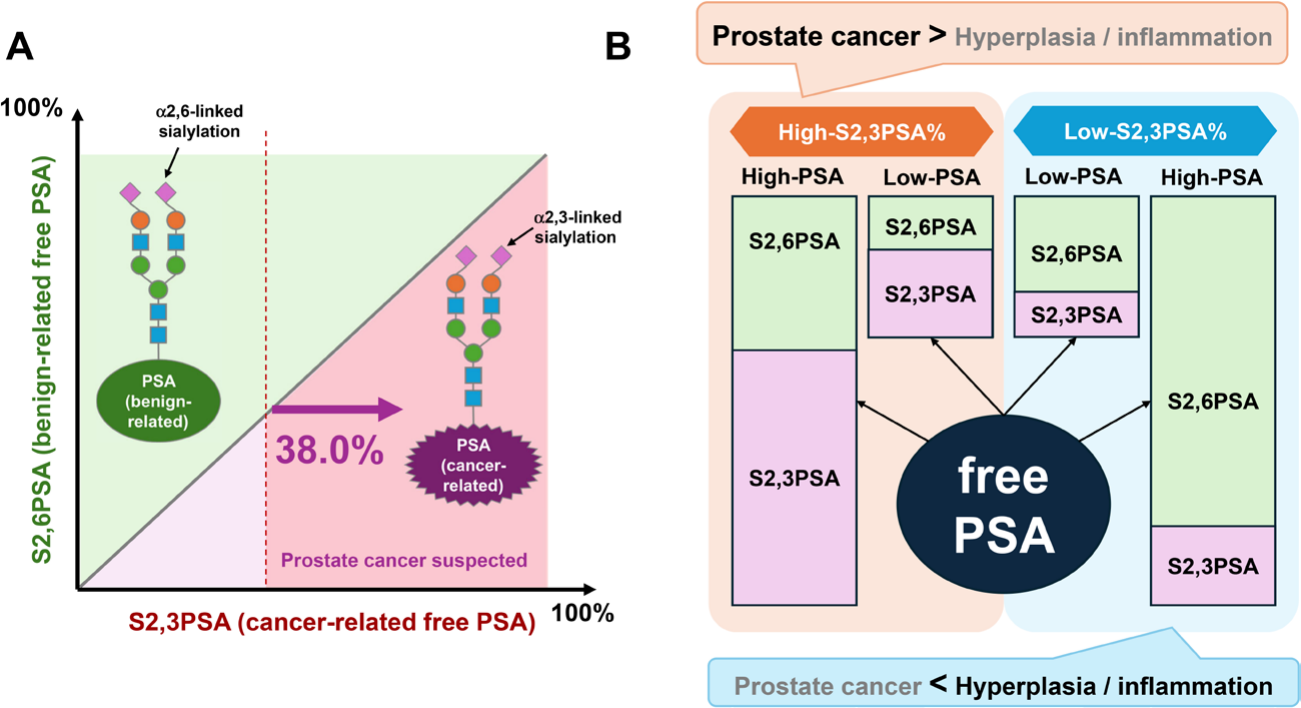
Conceptual framework of S2,3PSA% based on cancer‐associated PSA glycosylation. (A) Conceptual representation of the relative distribution of cancer‐related and benign‐related free PSA glycoforms. S2,6‐linked PSA predominantly reflects benign conditions, whereas S2,3‐linked PSA is enriched in prostate cancer–associated PSA. The diagonal boundary illustrates the balance between these two glycoforms, and an increased proportion of S2,3PSA shifts samples toward the cancer‐suspected region. A representative cutoff of 38% for S2,3PSA% is shown to distinguish cancer‐predominant from benign‐predominant PSA profiles. (B) Schematic illustration of the biological rationale for S2,3PSA% as a ratio‐based biomarker derived from free PSA. In prostate cancer, the relative proportion of S2,3‐linked PSA increases compared with S2,6‐linked PSA, regardless of absolute PSA levels, resulting in a high S2,3PSA%. In contrast, benign conditions such as prostatic hyperplasia or inflammation are characterized by a predominance of S2,6‐linked PSA, leading to a low S2,3PSA%. This ratio‐based approach enables discrimination of cancer‐associated PSA glycosylation patterns independent of total PSA concentration.

Figure 1A schematically illustrates the conceptual relationship between benign‐associated and cancer‐associated PSA glycoforms based on their sialylation patterns. PSA derived predominantly from benign prostate tissue is enriched with S2,6PSA, whereas PSA originating from prostate cancer shows a relative predominance of S2,3PSA. Rather than representing absolute quantities, the figure emphasizes the balance between these two PSA glycoforms, highlighting a shift from a benign‐dominant to a cancer‐dominant glycosylation profile. As the proportion of S2,3PSA increases relative to S2,6PSA, the biological likelihood of prostate cancer correspondingly increases. This conceptual framework provides the biological basis for distinguishing cancer‐related PSA from benign‐related PSA and sets the stage for ratio‐based evaluation of PSA glycoforms (Figure 1A).

### 2.2 Analytical Development of S2,3PSA% Within the Free PSA Fraction

Translating cancer‐associated PSA glycosylation into a clinically applicable biomarker required the establishment of a robust, reproducible, and automated analytical platform. Early lectin‐based immunoassays demonstrated the feasibility of selectively detecting S2,3PSA within the free PSA fraction; however, their initial formats were labor‐intensive and not suitable for clinical use [40].

To overcome these limitations, an automated microfluidic immunoassay system (microTAS Wako system) was developed and validated. This platform integrates lectin capillary electrophoresis‐based immunoassays within a fully automated microchip‐based analyzer, enabling high‐throughput and standardized measurement of S2,3PSA% [41]. The microTAS Wako system minimizes operator‐dependent variability and allows simultaneous quantification of total PSA and α2,3‐linked PSA glycoforms from small serum volumes.

**FIGURE 2:**
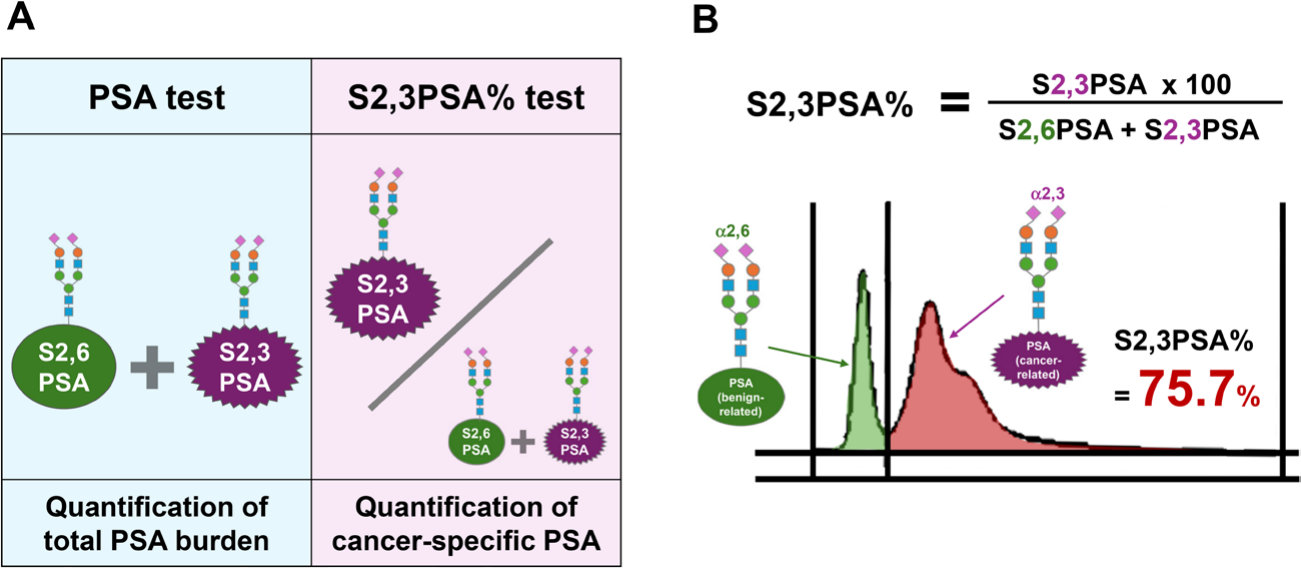
Analytical concept and calculation of S2,3PSA%. (A) Conceptual comparison between conventional PSA testing and the S2,3PSA% assay. Standard PSA testing quantifies the total amount of free PSA without distinguishing between different glycoforms, thereby providing primarily (quantitative). In contrast, the S2,3PSA% test qualitatively evaluates the relative composition of cancer‐associated (S2,3‐linked) and benign‐associated (S2,6‐linked) PSA glycoforms within the free PSA fraction (Qualitative). By assessing the balance between these two glycosylation patterns rather than absolute PSA concentration, S2,3PSA% emphasizes cancer‐related PSA characteristics. (B) Schematic illustration of the calculation and interpretation of S2,3PSA%. S2,3PSA% is defined as the proportion of S2,3‐linked PSA relative to the sum of S2,3‐linked and S2,6‐linked PSA, expressed as a percentage. Representative signal distributions demonstrate a predominance of S2,6‐linked PSA in benign conditions and enrichment of S2,3‐linked PSA in prostate cancer–associated PSA, resulting in a high S2,3PSA% value (example shown: 75.7%). This ratio‐based approach enables discrimination of cancer‐associated PSA glycosylation patterns independent of total PSA levels.

A critical conceptual feature of S2,3PSA% is the use of a ratio‐based metric rather than absolute concentrations of individual PSA glycoforms within the free PSA fraction. Quantification of S2,3PSA or S2,6PSA alone is inherently influenced by total PSA levels, which vary widely according to benign prostatic hyperplasia, inflammation, and prostate volume (Figure 1B). As a result, absolute glycoform values may still reflect overall PSA burden rather than cancer‐specific biological characteristics. To overcome this limitation, S2,3PSA% was designed to express the proportion of cancer‐associated α2,3‐linked PSA relative to the total PSA glycoform pool, thereby minimizing confounding by total PSA concentration and emphasizing qualitative differences in PSA glycosylation (Figure 2A). Specifically, S2,3PSA% is calculated as the ratio of S2,3PSA to the sum of S2,3PSA and S2,6PSA, multiplied by 100 (S2,3PSA% = [S2,3PSA/(S2,6PSA + S2,3PSA)] × 100). This formulation enables normalization of cancer‐associated PSA glycoforms against benign‐associated PSA glycoforms within the same serum sample. The resulting S2,3PSA% value directly reflects the relative dominance of cancer‐associated PSA glycosylation, independent of absolute PSA concentration (Figure 2B). By adopting this ratio‐based approach, S2,3PSA% provides a robust and biologically meaningful indicator of prostate cancer–related PSA glycosylation patterns that is less susceptible to interindividual variability in total PSA levels.

Analytical validation studies demonstrated excellent reproducibility and measurement stability. Intra‐assay and inter‐assay coefficients of variation were consistently low, indicating high precision across repeated measurements. Importantly, S2,3PSA% values remained stable across clinically relevant PSA concentration ranges (4–50 ng/mL), supporting the robustness of ratio‐based normalization [51]. Stability testing further confirmed that S2,3PSA% measurements were not substantially affected by short‐term sample storage or routine handling conditions [44]. Specifically, S2,3PSA% remained stable in whole blood for up to 24 h after collection, in serum stored at 4°C for up to 7 days, and in serum stored at −20°C for at least 1 month. In addition, repeated freeze–thaw cycles (up to 5 cycles) did not result in clinically meaningful changes in S2,3PSA% values. These findings indicate that S2,3PSA% is analytically robust under clinical laboratory conditions, supporting its feasibility for real‐world implementation.

The analytical performance of the microTAS Wako system was comprehensively reported in a validation study [41, 44]. This study established that automated measurement of S2,3PSA% is technically feasible, analytically reliable, and suitable for clinical research and potential routine use. By enabling standardized assessment of cancer‐associated PSA glycoforms, the microTAS Wako system provided a critical technological foundation for subsequent clinical studies evaluating the diagnostic utility of S2,3PSA%.

## 3 Early Clinical Evidence Before MRI Integration

### 3.1 Diagnostic Performance in the PSA Gray Zone

Initial clinical investigations evaluated S2,3PSA% in patients within the PSA gray zone, typically defined as PSA levels between 4 and 10 ng/mL. In this population, S2,3PSA% demonstrated superior discrimination of csPC compared with total PSA alone [41, 51]. Importantly, elevated S2,3PSA% values were associated with higher Gleason grade and adverse pathological features. These early studies established the clinical relevance of PSA glycosylation patterns and provided proof‐of‐concept that S2,3PSA% could improve risk stratification in patients traditionally considered diagnostically ambiguous.

### 3.2 Comparison With Established PSA‐Based Biomarkers

Subsequent analyses compared S2,3PSA% with established PSA derivatives, including %fPSA and PHI. While PHI integrates PSA isoforms to enhance diagnostic accuracy, S2,3PSA% reflects a fundamentally different biological dimension. Studies consistently showed that S2,3PSA% retained independent predictive value for csPC when adjusted for PHI, indicating that it provides additional biological information beyond PHI [41, 51]. These findings indicated S2,3PSA% as a qualitative biomarker that adds biological specificity beyond PSA concentration–based indices. The study provided early clinical evidence that S2,3PSA% captures prostate cancer–specific biological information beyond total PSA, forming the basis for later integration with MRI‐based diagnostic strategies [51].

### 3.3 Risk Stratification by S2,3PSA% Independent of PSA Levels

**FIGURE 3:**
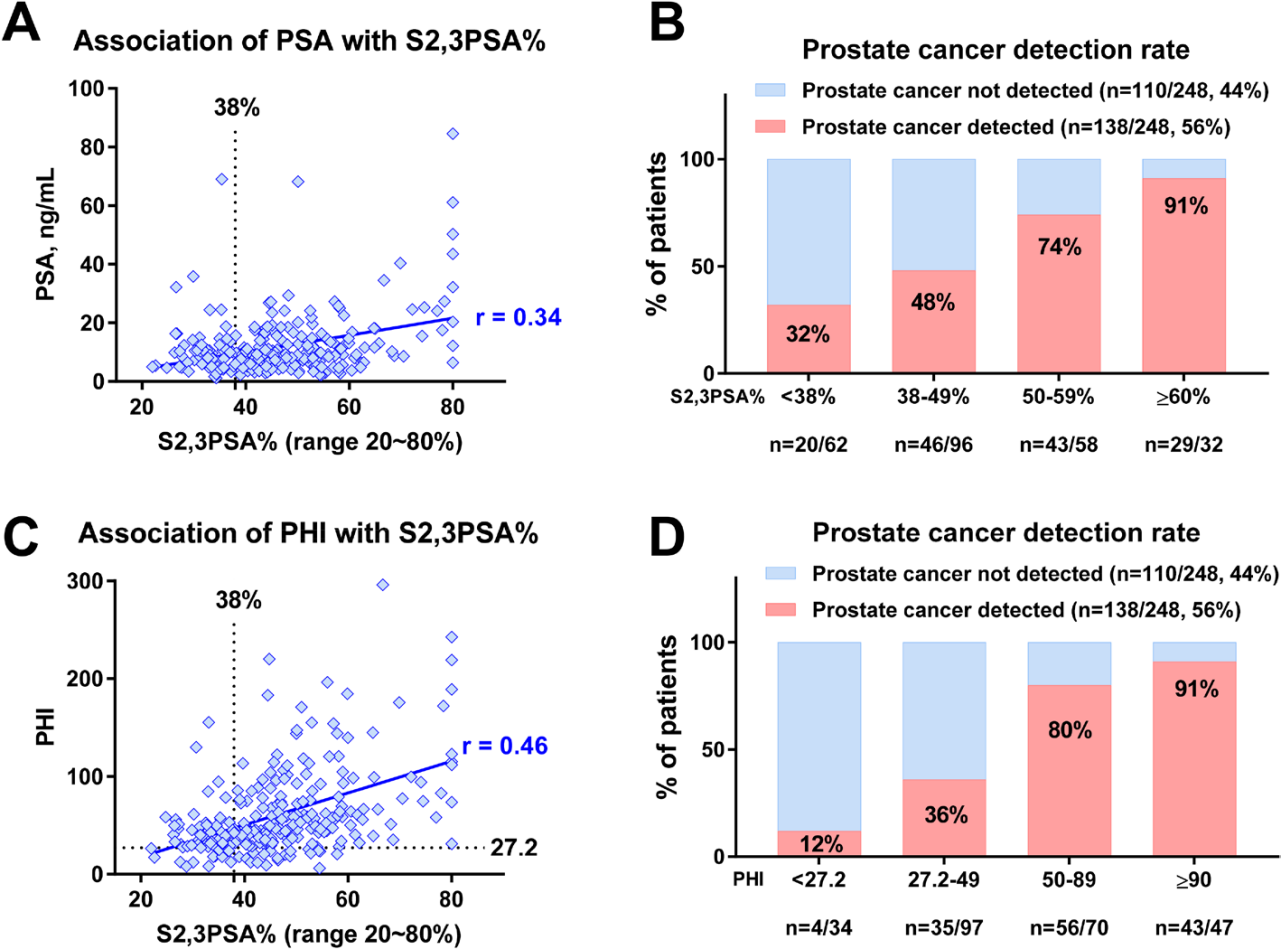
Relationship between PSA levels, S2,3PSA%, and prostate cancer detection. (A) Scatter plot showing the association between total PSA concentration and S2,3PSA%. The correlation between PSA and S2,3PSA% was weak (*r* = 0.32), indicating that S2,3PSA% provides biological information largely independent of PSA quantity. The dashed vertical line represents a representative cutoff of 38% for S2,3PSA%, highlighting variability in S2,3PSA% across a wide range of PSA values. (B) Prostate cancer detection rates stratified by S2,3PSA% categories in a biopsy cohort (*n* = 207). Cancer detection rates increased stepwise with higher S2,3PSA% levels, from 36% in patients with S2,3PSA% < 38%–100% in those with S2,3PSA% ≥ 65%. Notably, prostate cancer was still detected in the lowest S2,3PSA% group, indicating that low S2,3PSA% does not exclude malignancy. These analyses were performed across a broad range of PSA values and were not restricted to the PSA gray zone.

Figure 3 illustrates the clinical relevance of S2,3PSA% stratification using data from a biopsy cohort of 248 men reported by Oishi et al. (median age 71 years [interquartile range 65–75]; median PSA 9.16 ng/mL [interquartile range 5.8–14.3]) [46]. The correlation between total PSA concentration and S2,3PSA% was weak (*r* = 0.34; Figure 3A), indicating that S2,3PSA% captures biological information largely independent of PSA quantity. This finding supports the concept that S2,3PSA% reflects qualitative differences in PSA glycosylation rather than simple PSA production.

Stratification by S2,3PSA% revealed a stepwise increase in prostate cancer detection rates with increasing values (Figure 3B). Notably, prostate cancer was still detected in patients with S2,3PSA% < 38%, highlighting that low S2,3PSA% does not exclude malignancy. This subgroup may include tumors with low PSA productivity, such as Gleason 5 disease. Importantly, however, the proportion of cancer‐positive cases increased progressively across higher S2,3PSA% categories, reaching a detection rate of 91% in patients with S2,3PSA% ≥ 60%. These trends were observed in a cohort not restricted to the PSA 4–10 ng/mL range, highlighting the robustness of S2,3PSA%–based risk stratification across a broad spectrum of PSA values.

### 3.4 Relationship Between S2,3PSA% and PHI and Complementary Risk Stratification

The relationship between S2,3PSA% and the PHI was further examined using data from a biopsy cohort of 248 men reported by Oishi et al. [46]. S2,3PSA% showed a moderate correlation with PHI (*r* = 0.46), which was stronger than that observed between S2,3PSA% and total PSA, yet still indicated that these biomarkers capture partially distinct biological information (Figure 3C). When stratified by PHI categories, prostate cancer detection rates increased stepwise with higher PHI values, consistent with prior reports (Figure 3D) [16, 17, 22]. Notably, prostate cancer was detected even in patients with low PHI values, highlighting the limitation of relying on a single biomarker. Collectively, these findings support the concept that S2,3PSA% and PHI reflect overlapping but nonredundant aspects of prostate cancer biology, providing a rationale for their combined use in multivariable diagnostic models.

### 3.5 Other Biomarker to Improve Risk Stratification

In the PSA gray zone, several other biomarkers have been proposed to improve risk stratification, including PSA density (PSAD), the 4Kscore panel, and the urine‐based molecular marker prostate cancer gene 3 (PCA3) [20]. PSA density adjusts serum PSA levels by prostate volume and is widely used in conjunction with MRI; however, it remains a quantitative measure influenced by benign prostatic enlargement and does not capture tumor‐specific biological alterations. Nevertheless, recent studies have suggested that PSA density may provide incremental diagnostic value when combined with other biomarkers or clinical parameters, particularly within multiparametric risk stratification models [35, 45, 52, 53, 54, 55, 56, 57, 58, 59, 60, 61, 62]. In contrast, S2,3PSA% reflects qualitative changes in PSA glycosylation associated with malignant transformation and provides information that is largely independent of prostate volume. Indeed, emerging evidence suggests that combining S2,3PSA% with PSA density may further improve risk stratification performance [45], supporting the clinical validity of PSA density–based strategies when integrated with tumor‐specific glycoform biomarkers.

The 4Kscore combines multiple kallikrein markers to estimate the probability of high‐grade prostate cancer [60, 63, 64], while PCA3 evaluates prostate cancer–associated gene expression in urine after digital rectal examination [65, 66]. Although both approaches have demonstrated clinical utility, they rely on different biological principles from S2,3PSA% and are not currently reimbursed in Japan, limiting their clinical use. At present, direct comparative studies between S2,3PSA% and these biomarkers are limited. Nevertheless, the distinct mechanistic basis of S2,3PSA% suggests that it may serve as a complementary blood‐based biomarker, particularly in settings where access to multi‐marker panels or urine‐based assays is restricted. Further comparative and integrative studies are warranted to clarify the optimal positioning of S2,3PSA% within multimodal diagnostic strategies.

## 4 S2,3PSA% in the MRI‐Guided Biopsy Era

### 4.1 Limitations of MRI‐Only Strategies

Although mpMRI has transformed prostate cancer diagnostics, reliance on imaging alone has limitations [34, 67, 68, 69]. PI‐RADS 3 lesions represent a major source of uncertainty, with variable csPC detection rates across institutions [25, 26, 27, 28, 29, 30, 31, 32, 33, 34, 35, 36]. Furthermore, MRI‐negative cases may still harbor csPC, while MRI‐positive findings can lead to unnecessary biopsies due to false‐positive interpretations. These challenges highlight the need for biomarkers that provide biological information complementary to MRI findings.

### 4.2 Integration of S2,3PSA% With MRI, With or Without PHI

**FIGURE 4:**
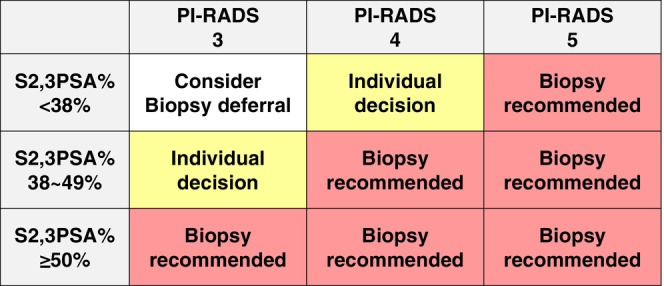
Conceptual clinical approach integrating S2,3PSA% with PI‐RADS categories for biopsy decision‐making. Schematic illustration of a proposed clinical approach integrating S2,3PSA% with MRI findings stratified by PI‐RADS category to guide prostate biopsy decisions. For intermediate S2,3PSA% (38%–49%), biopsy decisions are refined by MRI findings, with individual decision for PI‐RADS 3 lesions and biopsy recommended for PI‐RADS 4–5 lesions. In patients with low S2,3PSA% (< 38%), biopsy deferral (observation) is suggested for PI‐RADS 3 lesions, while individual decision or biopsy is recommended for PI‐RADS 4 and 5 lesions, respectively. Importantly, individual decision or biopsy deferral (observation) does not imply the absence of prostate cancer but indicates that immediate biopsy may be deferred with careful follow‐up. This hypothesis‐generating approach requires prospective validation.

Multiple real‐world cohorts have evaluated the role of S2,3PSA% in MRI‐targeted biopsy settings [19, 43, 44, 45, 46, 50]. These studies demonstrated that incorporating S2,3PSA% into MRI‐based clinical decision‐making reduced unnecessary biopsies without compromising csPC detection. Based on these accumulating findings, a conceptual clinical flowchart integrating S2,3PSA% with PI‐RADS categories was developed to support biopsy decision‐making in the MRI era (Figure 4). This proposed approach illustrates how S2,3PSA% may be used to refine biopsy recommendations by identifying patients suitable for immediate biopsy, shared decision‐making, or biopsy deferral (observation) according to combined biomarker and imaging risk profiles. Although prospective validation of this algorithm is still required, available real‐world data suggest that application of such an approach could avoid approximately 20% of prostate biopsies without compromising the detection of clinically significant prostate cancer. Accordingly, this flowchart should be regarded as a practical, hypothesis‐generating model that reflects current evidence and highlights the potential clinical impact of integrating S2,3PSA% into MRI‐guided biopsy strategies. Importantly, categories labeled as “individual decision” or “consider biopsy deferral” do not imply the absence of prostate cancer; rather, they indicate clinical scenarios in which immediate biopsy may be deferred and careful follow‐up is considered appropriate.

Furthermore, recent studies have focused on integrating S2,3PSA% with PHI and MRI findings [44, 45]. In MRI fusion biopsy cohorts, the combined use of S2,3PSA% and PHI improved discrimination of csPC compared with either biomarker alone. Importantly, S2,3PSA% showed utility in refining risk among patients with intermediate MRI findings.

### 4.3 S2,3PSA Density as an Extension of the Concept

The most recent evolution of this biomarker paradigm is the introduction of S2,3PSA density, which normalizes S2,3PSA% to prostate volume. In patients with PI‐RADS 3–5 lesions, S2,3PSA density further improved risk stratification beyond MRI alone. This approach extends the concept of PSA density to the glycoform level and represents a next‐generation refinement of biomarker‐guided biopsy decision‐making [44, 45].

### 4.4 Role of S2,3PSA% in Prostate Cancer Screening and Biopsy Triage

**FIGURE 5:**
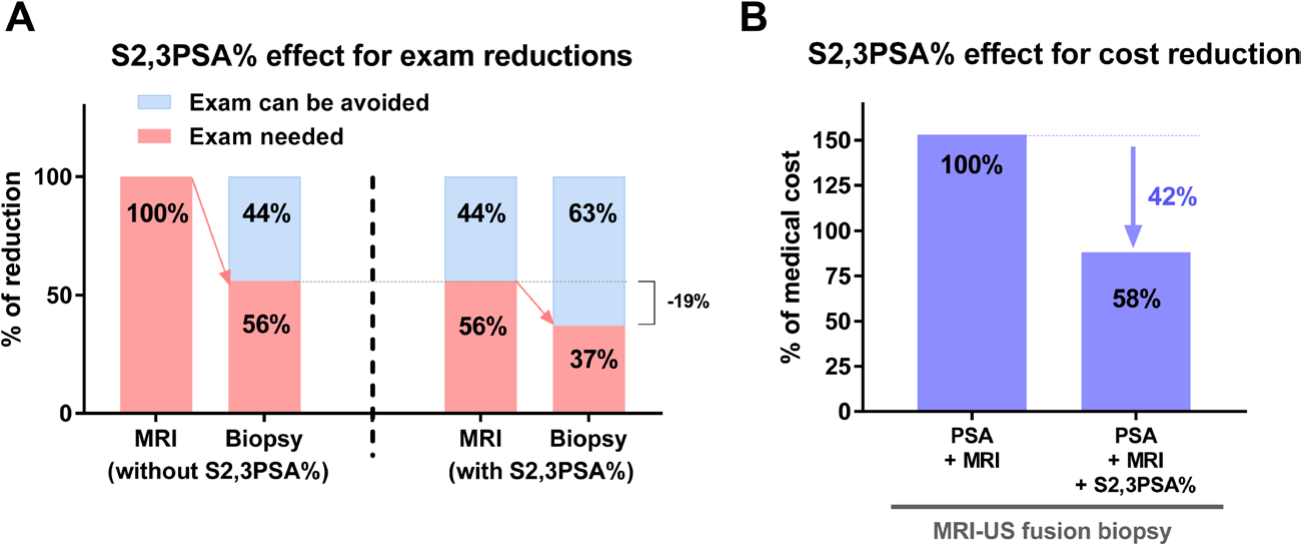
Impact of S2,3PSA% on screening‐based biopsy triage and healthcare costs. (A) Effect of incorporating S2,3PSA% into prostate cancer screening–based triage prior to MRI. Use of S2,3PSA% enabled stratification of biopsy risk before imaging, resulting in a reduction in unnecessary MRI examinations (44% reduction) and subsequent prostate biopsies (63% reduction) compared with strategies not incorporating S2,3PSA%. Blue bars indicate examinations that could be avoided, whereas red bars represent examinations still required. Avoidance of biopsy in patients with low S2,3PSA% does not indicate absence of prostate cancer but reflects a subgroup in whom immediate biopsy may be deferred with appropriate follow‐up. (B) Conceptual impact of S2,3PSA%–guided screening on healthcare costs. Incorporation of S2,3PSA% into PSA‐ and MRI‐based screening pathways was associated with a substantial reduction in overall medical costs related to MRI–ultrasound fusion biopsy, with an estimated cost reduction of approximately 42% compared with PSA plus MRI alone. This illustrates the potential economic benefit of S2,3PSA%–guided risk‐adapted screening strategies.

In addition to its role in MRI‐targeted biopsy cohorts, the clinical utility of S2,3PSA% has also been evaluated in the context of prostate cancer screening. In a recent screening‐based study [43], S2,3PSA% was shown to effectively stratify biopsy risk prior to MRI, leading to a reduction in unnecessary MRI examinations (44% reduction) and subsequent prostate biopsies (63% reduction) (Figure 5A). By identifying individuals with a low likelihood of clinically significant prostate cancer, S2,3PSA% enabled deferral of immediate imaging and invasive procedures without compromising overall risk assessment. Importantly, avoidance of biopsy in patients with low S2,3PSA% does not imply the absence of prostate cancer; rather, it suggests a subgroup in whom “immediate biopsy may not be required” and careful longitudinal follow‐up is clinically acceptable. From a practical standpoint, this risk‐adapted approach aligns with real‐world screening strategies, in which the timing of biopsy is often as important as the decision to biopsy itself. Furthermore, by reducing the number of unnecessary MRI scans and biopsies, incorporation of S2,3PSA% into screening algorithms has the potential to lower healthcare costs (42% reduction) and optimize resource utilization (Figure 5B), highlighting its value not only in clinical decision‐making but also in healthcare economics.

## 5 Integrated Risk Models and Decision‐Making

### 5.1 Nomogram‐Based Approaches

Building on these findings, nomogram‐based models integrating age, PHI, S2,3PSA%, and PI‐RADS category have been developed to provide individualized risk estimates for csPC. In a representative MRI‐targeted biopsy cohort, multivariable logistic regression identified age, S2,3PSA%, and PI‐RADS category as independent predictors of csPC, while PHI contributed complementary predictive information. The resulting nomogram demonstrated excellent apparent discrimination (AUC 0.86), and near‐ideal calibration (calibration slope 0.91, low Brier score). Importantly, decision curve analyses consistently showed a superior net clinical benefit across clinically relevant threshold probabilities compared with MRI‐only or single‐biomarker strategies. From a clinical perspective, these nomogram‐based approaches enable continuous, patient‐specific risk estimation rather than reliance on dichotomous cutoffs. This is particularly valuable in patients with PI‐RADS 3–4 lesions, where biomarker‐informed risk gradients meaningfully influence biopsy decision‐making. Net reduction analyses further suggested that such models could avoid approximately 33.9 unnecessary biopsies per 100 men, depending on the selected risk threshold, without substantially increasing missed csPC cases.

### 5.2 Beyond the Nomogram: Simplified Model‐Based Approaches

**FIGURE 6:**
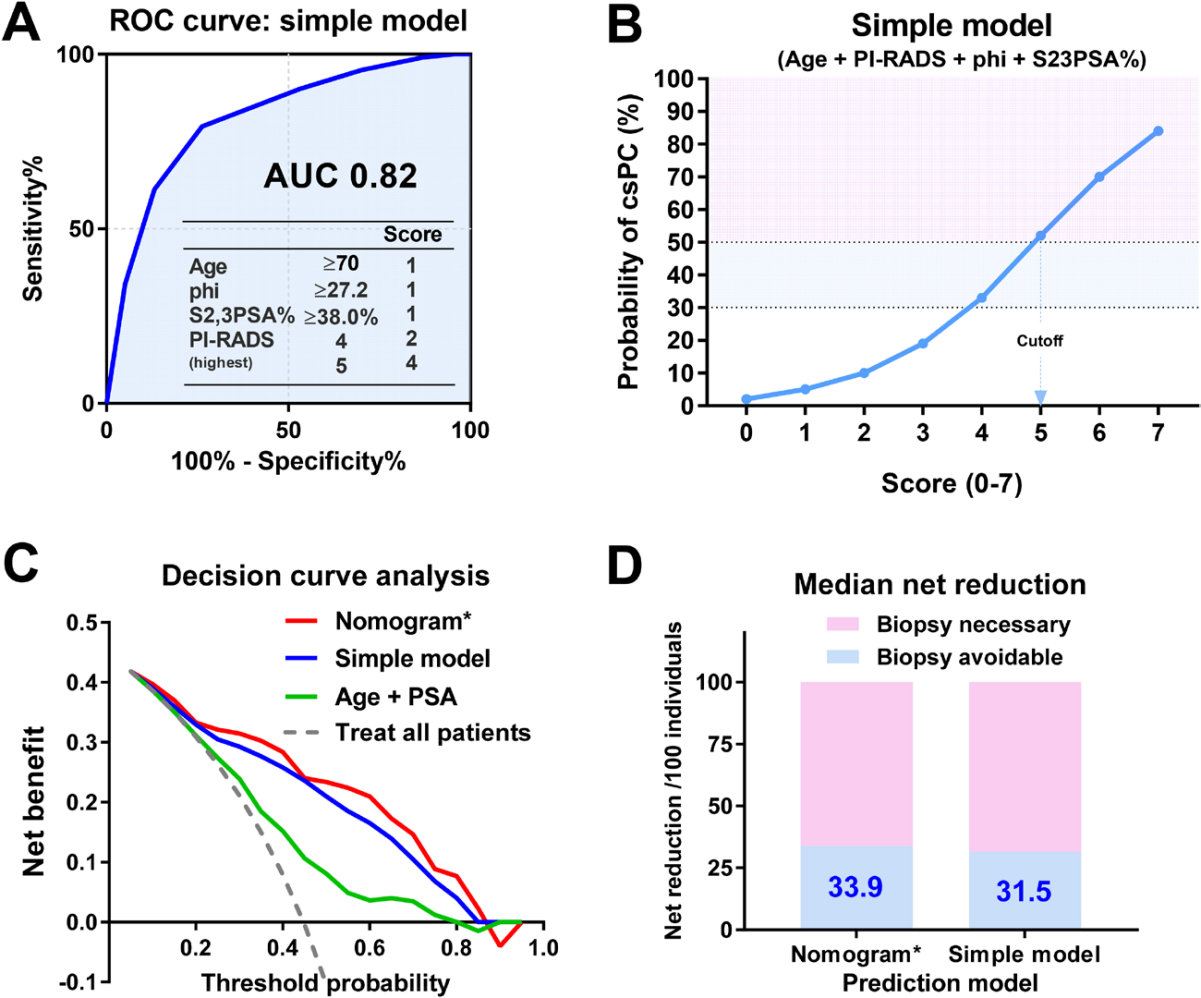
Performance of a simplified risk model integrating S2,3PSA% with MRI and PSA‐related markers. (A) Receiver operating characteristic (ROC) curve of a simplified risk model incorporating age, PI‐RADS category, Prostate Health Index (PHI), and S2,3PSA%. The model demonstrated good discriminative performance with an area under the curve (AUC) of 0.82, comparable to that of a full nomogram‐based model*. (B) Estimated probability of clinically significant prostate cancer (csPC) according to the simplified risk score (range, 0–7). A cutoff score of ≥ 5 corresponded to a predicted csPC probability exceeding 50%, providing a clinically intuitive threshold for biopsy consideration. (C) Decision curve analysis comparing the net benefit of the simplified model with that of the nomogram‐based model, age plus PSA, and a treat‐all strategy across a range of threshold probabilities. The simplified model demonstrated net benefit profiles similar to those of the nomogram. (D) Median net reduction in unnecessary biopsies per 100 individuals achieved by the nomogram‐based model* and the simplified model. The simplified model avoided 31.5 unnecessary biopsies per 100 men, only slightly fewer than the nomogram (33.9 per 100 men). *Detailed data for the nomogram‐based model are reported in Oishi T et al., *European Urology Open Science* 2026; 83: 158–65.

Despite their strong predictive performance, full nomogram‐based tools may be cumbersome to apply in clinical practice. To improve usability, a simplified risk model incorporating four key variables—age, PHI, S2,3PSA%, and PI‐RADS category—was therefore developed (Table 1). This simple model showed similar discriminatory power to the original nomogram (AUC 0.82; Figure 6A), indicating minimal loss of predictive accuracy despite substantial simplification. In this scoring system, a cutoff value of ≥ 5 corresponded to an estimated probability of clinically significant prostate cancer exceeding 50% (Figure 6B), providing an intuitive threshold for biopsy consideration. Decision curve analysis showed that the simplified model achieved net benefit profiles similar to those of the nomogram (Figure 6C), with a median net reduction of 31.5 unnecessary biopsies per 100 men—only slightly lower than that of the nomogram (33.9 per 100 men) (Figure 6D). Collectively, these findings suggest that simplified, clinically accessible models incorporating S2,3PSA% retain most of the decision‐making advantages of more complex nomogram‐based approaches, while offering greater practicality for real‐world implementation. Nevertheless, external validation in multicenter cohorts remains essential before widespread adoption.

**TABLE 1:** Multivariable logistic regression analysis for csPC (data from [46]).

Model for nomogram	Standard regression coefficient	*p* value	Odds ratio	95% CI
Age	0.63	< 0.001	1.10	1.04–1.05
PHI	0.38	0.076	1.01	1.00–1.05
S2,3PSA%	0.74	< 0.001	1.06	1.03–1.09
Highest PI‐RADS	0.97	< 0.001	2.16	2.16–5.59

Model for simple model	Standard regression coefficient	*p* value	Odds ratio	95% CI
Age ≥ 70	0.46	0.004	2.53	1.35–4.73
PHI ≥ 27.2	0.61	0.008	5.91	1.59–21.9
S2,3PSA% ≥ 38.0%	0.45	0.007	2.85	1.34–6.06
Highest PI‐RADS	1.06	< 0.001	3.94	2.52–6.16

## 6 Timeline‐Based Diagnostic Strategy Incorporating S2,3PSA%

**FIGURE 7:**
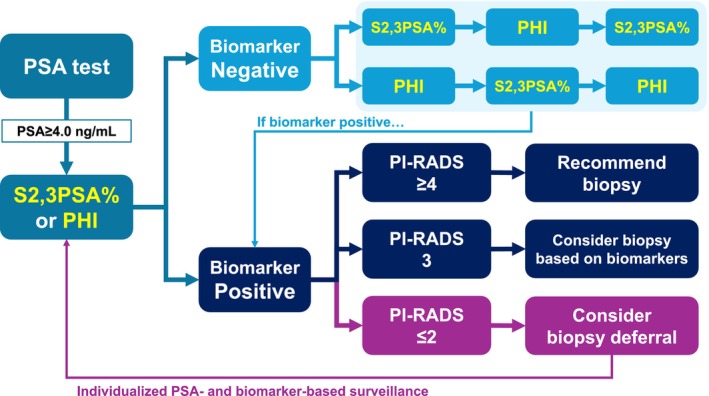
Timeline‐based diagnostic flowchart integrating S2,3PSA% into prostate cancer evaluation. Following initial PSA testing, S2,3PSA% or PHI may be measured in patients with PSA ≥ 4.0 ng/mL to refine pre‐imaging risk stratification. Biomarker‐positive patients proceed to multiparametric MRI, with biopsy decisions guided by PI‐RADS category.

To facilitate clinical implementation, we summarize a simplified, timeline‐based diagnostic pathway integrating S2,3PSA% into contemporary prostate cancer assessment (Figure 7). Following initial total PSA testing, patients with PSA ≥ 4.0 ng/mL may undergo secondary biomarker evaluation using either S2,3PSA% or the PHI. These biomarkers can be measured interchangeably or sequentially, depending on availability and clinical context, to refine risk assessment before imaging.

Patients with biomarker‐negative results may enter individualized PSA‐ and biomarker‐based surveillance, whereas biomarker‐positive findings prompt multiparametric MRI. Subsequent biopsy decisions are guided by PI‐RADS category: biopsy is recommended for PI‐RADS ≥ 4, considered after shared decision‐making for PI‐RADS 3, and may be deferred for PI‐RADS ≤ 2 with continued surveillance. This stepwise approach highlights how S2,3PSA% can be applied early in the diagnostic timeline to reduce unnecessary MRI examinations and biopsies, while preserving timely detection of clinically significant prostate cancer. Importantly, this framework reflects current evidence and is intended as a practical, hypothesis‐generating model rather than a prescriptive guideline.

## 7 Role in Active Surveillance

### 7.1 Predicting Reclassification Risk During Active Surveillance

Emerging evidence suggests that S2,3PSA% may also have a role in the longitudinal management of patients undergoing active surveillance. In a recent longitudinal analysis, higher S2,3PSA% levels were associated with an increased risk of disease reclassification over time, indicating potential progression beyond initial low‐risk features. These findings imply that S2,3PSA% may serve as a dynamic biomarker capable of capturing biological changes related to tumor aggressiveness that are not fully reflected by conventional clinical parameters alone [42]. Importantly, the ability to predict reclassification risk using a blood‐based marker could complement existing surveillance protocols by informing the intensity and timing of follow‐up evaluations, including repeat biopsy or imaging. Although these observations are currently based on early‐phase data and require validation in larger, prospective cohorts, they highlight the potential utility of S2,3PSA% as a noninvasive tool for risk‐adapted monitoring during active surveillance.

## 8 Limitations and Unmet Needs

Despite encouraging evidence, several limitations and unmet needs remain regarding the clinical application of S2,3PSA%. Most available studies are retrospective and single‐center, highlighting the need for prospective validation and external confirmation in multicenter and ethnically diverse populations. In addition, further standardization of assay methodology, cutoff values, and comprehensive cost‐effectiveness analyses are required before widespread implementation. An important limitation is that S2,3PSA% is derived from the free PSA fraction, which restricts its utility at very low PSA levels. When total PSA is below approximately 2 ng/mL, reliable assessment becomes difficult, limiting its applicability in certain clinical scenarios. Accordingly, S2,3PSA% is unlikely to be informative for biochemical recurrence after radical prostatectomy, where PSA levels typically remain below the range suitable for free PSA–based analyses. Moreover, its role in advanced disease states, including castration‐resistant prostate cancer, has not yet been systematically investigated. Conversely, this PSA threshold also defines potential future applications. Because S2,3PSA% remains informative at PSA levels above 2 ng/mL, it may be useful in post‐radiation settings, where PSA bounce complicates clinical interpretation. In addition, following focal therapies for prostate cancer, S2,3PSA% may provide complementary information on residual or recurrent disease beyond absolute PSA kinetics. Exploratory investigations in these settings have begun, but robust clinical validation is still required. Overall, while current evidence supports the role of S2,3PSA% primarily in diagnosis and surveillance, further studies are needed to clarify its applicability across the broader spectrum of prostate cancer.

## 9 Conclusions

S2,3PSA% represents a biologically informed biomarker that addresses persistent diagnostic challenges in prostate cancer detection. By capturing cancer‐associated differences in PSA glycosylation, S2,3PSA% adds useful information beyond PSA tests and MRI. Accumulating evidence supports its role in reducing unnecessary prostate biopsies while maintaining detection of clinically significant disease. Integration of S2,3PSA% into contemporary diagnostic pathways may help clarify persistent diagnostic uncertainty in PSA‐based prostate cancer screening and advance personalized prostate cancer care.

## Author Contributions

**Shingo Hatakeyama:** conceptualization, investigation, funding acquisition, writing – original draft, methodology, validation, visualization, writing – review and editing, software, formal analysis, project administration, data curation, resources. **Tohru Yoneyama:** supervision, resources, conceptualization, project administration, data curation, funding acquisition. **Chikara Ohyama:** supervision, resources, conceptualization, project administration, data curation.

## Funding

The Japan Society for the Promotion of Science (JSPS) KAKENHI (grant number, 25 K02769; Shingo Hatakeyama and 24 K12501; Tohru Yoneyama).

## Ethics Statement

This study was conducted in accordance with the Declaration of Helsinki. The study was approved by the Ethics Committee of the Hirosaki University School of Medicine (2019‐099‐3, 2022‐140‐2, and 2023‐029) and all the hospitals involved in the study.

## Consent

The study information was publicly disclosed in lieu of written informed consent, and patients could opt out of the study (opt‐out approach).

## Conflicts of Interest

Shingo Hatakeyama received honoraria from Janssen Pharmaceutical K.K., Astellas Pharma Inc., AstraZeneca K.K., Ono Pharmaceutical Co. Ltd., Bayer AG, Pfizer Inc., Bristol‐Myers Squibb, Merck Biopharma Co. Ltd., Kaneka Corporation, and Nipro Corporation. The other authors have no conflicts of interest to declare. Shingo Hatakeyama is an Editorial Board member of the International Journal of Urology and a co‐author of this article. To minimize bias, they were excluded from all editorial decision‐making related to the acceptance of this article for publication.

## Data Availability

The data that support the findings of this study are available from the corresponding author upon reasonable request.
